# Medicinal Plants in the Treatment of Myocardial Infarction Disease: A Systematic Review

**DOI:** 10.2174/011573403X278881240405044328

**Published:** 2024-04-29

**Authors:** Anamika Rathore, Anuj Kumar Sharma, Yogesh Murti, Sonal Bansal, Vibha Kumari, Varsha Snehi, Mayank Kulshreshtha

**Affiliations:** 1 Department of Pharmacology, Rajiv Academy for Pharmacy, Mathura, Uttar Pradesh, India;; 2 G.L.A. University, Mathura, Uttar Pradesh, India;; 3 Department of Pharmaceutical Chemistry, Rajiv Academy for Pharmacy, Mathura, Uttar Pradesh, India;; 4 Department of Pharmaceutics, Rajiv Academy for Pharmacy, Mathura, Uttar Pradesh, India

**Keywords:** Myocardial infarction, phytochemicals, *Fabaceae*, coronary artery, glucosides, alkaloids

## Abstract

**Background:**

Myocardial infarction (MI), also referred to as a “heart attack,” is brought on by a partial or total interruption of blood supply to the myocardium. Myocardial infarction can be “silent,” go undiagnosed, or it can be a catastrophic occurrence that results in hemodynamic decline and untimely death. In recent years, herbal remedies for MI have become effective, secure, and readily accessible.

**Objective:**

The purpose of this review was to examine the medicinal plants and phytochemicals that have been used to treat MI in order to assess the potential contribution of natural substances to the development of herbal MI treatments.

**Methodology:**

A literature search was employed to find information utilizing electronic databases, such as Web of Science, Google Scholar, PubMed, Sci Finder, Reaxys, and Cochrane.

**Results:**

The identification of 140 plants from 12 families led to the abstraction of data on the plant families, parts of the plant employed, chemical contents, extracts, model used, and dose.

**Conclusion:**

The majority of the MI plants, according to the data, belonged to the Fabaceae (11%) and Asteraceae (9%) families, and the most prevalent natural components in plants with MI were flavonoids (43%), glucosides (25%), alkaloids (23%), phenolic acid (19%), saponins (15%), and tannins (12%).

## 1 INTRODUCTION

### 1.1 Myocardial Infarction

Myocardial cells in myocardial infarction (MI) syndrome perish as a result of an imbalance between myocardial oxygen supply and demand. Type 1 myocardial infarction (T1MI) or type 2 myocardial infarction (T2MI) can both be attributable to acute atherosclerotic plaque disruption or changes in myocardial oxygen supply and/or demand in the absence of acute atherothrombosis [1]. It is a word used to describe a wide range of illnesses, including angina and ST-segment elevation myocardial infarction [2]. An atherosclerosis process that affects the coronary vasculature is the hallmark of coronary artery disease (CAD). Infants can show the initial signs of atherosclerosis, which then progresses through time with regression in childhood, recurrence in puberty, and other factors [3]. About 38.0% of Americans experience ST-elevation myocardial infarction (STEMI) on an annual basis [4], while white people living in the UK have a 50 times lower risk of MI than South Asians do [5]. Although STEMI typically only affects patients over the age of 45, its prevalence in younger people may raise the risk of premature death and result in long-term disability [6, 7]. Heart attacks, commonly known as myocardial infarctions (MI), are among the most prevalent chronic non-communicable diseases that cannot be passed from one person to another. It advances gradually and has an impact on the blood flow of the . A critical medical emergency called a myocardial infarction happens when one of the arteries delivering blood to the heart becomes blocked. Due to this, low blood oxygen levels in the heart produce recognisable chest discomfort and myocardial tissue loss. Diabetic patient has a 2-4 times more increased risk in developing MI as compared to the non-diabetic population [8]. There are four possible categories of the causes of MI in individuals under 45:

· Non-atheromatous CHD
· Atheromatous CHD
· MI related to substance misuse
· Hypercoagulable states

The prevalence of myocardial infarction varies widely over the globe. Nearly 650.000 and 180.000 persons, respectively, experience an acute myocardial infarction each year in the United States and the United Kingdom [9]. Over 3 million people worldwide have ST elevation myocardial infarction, and 4 million have Non-ST-elevation myocardial infarction [10].

Indians are four times as likely to get AMI due to a confluence of hereditary and lifestyle factors that encourage metabolic dysregulation compared to the inhabitants of other nations [11]. In India, there are 64.37 myocardial infarctions per 1000 individuals. Myocardial infarction has a mortality rate of about 30%, and 1 in 25 individuals who survive their initial hospitalization pass away within the first year of their acute myocardial infarction (AMI). Myocardial infarction is the cause of 31.7% of deaths in India. In India, the prevalence of cardiovascular illnesses grew from 7% in 1970 to 32% in 2011 [12]. Some medications and chemicals can induce MI, and their methods can vary greatly, from having a direct harmful effect on cardiomyocytes to vasospasm, which is probably the most typical mechanism. There are instances of MI brought on by chemotherapy [13], triptans, an anti-migraine medication [14], and antibiotics (cefuroxime, amoxicillin with clavulanic acid) [15, 16]. Further, with 7.4 million deaths per year, CAD is the biggest cause of mortality, accounting for 13.2% of all deaths worldwide [17]. In wealthy nations, both the incidence of MI and STEMI patients has been declining over the past few years. It happens in about 66/100000 countries in Europe every year. Despite this, there are somewhat more patients who experience non-ST segment elevation myocardial infarction (132/100000/year) [18, 19]. Plasma proteins, blood cells, and flow all play a role in the dynamic process of thrombus development [20].

Thus, it is well-known that MI is the most severe manifestation of coronary heart disease and the leading cause of death worldwide. Pathologically, MI is defined as cardiac cell death due to acute or persistent coronary ischemia and hypoxia. Following MI, the loss of cardiomyocytes causes ventricular pathological remodelling, reduced myocardial contractile function, and, ultimately, heart failure. An infarcted heart exhibits the loss of cardiomyocytes and fibrotic scarring in the myocardium [21, 22].

### 1.2 Importance of Medicinal Plant

The term “medical plants” refers to a variety of plant species employed in herbalism, some of which have medicinal properties. These medicinal plants are regarded as a rich source of components for the creation and synthesis of medications. Additionally, these plants are essential to the growth of human cultures all across the world. Additionally, some plants are regarded as significant sources of nutrition, and as a result, these plants are suggested for their medicinal benefits. These plants include walnuts, ginger, green tea, and a few others. Other plants and their derivatives are regarded as key sources of the active compounds used in toothpaste and aspirin [23]. The therapeutic potential of plant products can be traced back more than 5,000 years since evidence suggests that Indian, Egyptian, Chinese, Greek, and Roman civilizations used them to treat illnesses and revitalise bodily systems [24]. Plants with medicinal potential are widely used in India by all socioeconomic groups as both traditional medicines in various indigenous medical systems like Siddha, Ayurveda, and Unani, as well as processed goods in the pharmaceutical sector [25]. It is possible to treat and cure diseases, including, myocardial infarction, ulcers, wounds, skin infections, inflammation, scabies, leprosy, and venereal disease with herbal medications [26]. Herbal remedies are used to clean wounds, remove dead tissue, and provide a moist environment that encourages the emergence of an ideal natural healing climate. Many plants are used in folklore cultures to cure burns, wounds, and cuts [27]. The leading cause of death worldwide is heart attack, often known as myocardial infarction (MI), and the complications that are associated with it [28]. The elements of the plants have been proven to be quite useful in the treatment of complicated instances like cancer disorders. Some plants are regarded as significant sources of nutrition, thereby exhibiting therapeutic properties.While others are known for their active compounds.

Additionally, normal wound healing involves a coordinated series of actions that start with an injury. The healing cascade is started when platelets come into touch with exposed collagen. This results in an accumulation of platelets and the release of coagulating substances, which leads to the creation of a fibrin clot at the injury site. The fibrin clot serves as a temporary matrix that directs the processes involved in healing [29].

Throughout history, humans have relied on nature to provide them with their basic needs, including food, housing, medicine, clothes, flavourings, fertilisers, and transportation. Through the ages, there have been changes in food, clothing, flavours, scents, fertilisers, and modes of transportation. This is especially true in developing nations, where herbal medicine has a long history of use and where medicinal plants continue to play a significant role in the healthcare system for vast segments of the world's population [30]. The use of traditional medicine and medicinal plants as a foundation for maintaining good health has been generally acknowledged by the United Nations Educational, Scientific, and Cultural Organization since 1996 [31]. Additionally, the growing reliance of the industrialised nations on the use of therapeutic herbs can be linked to the several medicines and chemotherapeutics that are extracted from these plants and developed, along with conventionally used rural herbal treatments [32]. Natural products are an important source of drug compounds, and many modern medications that are developed from conventional herbal medicine are presently used in modern pharmacotherapy [33]. The future of medicinal plants is bright because there are around 500,000 plants around the globe, and the medical properties of the majority of them have not yet been explored. These properties could be crucial in the treatment of ongoing or upcoming studies. An important step in the investigation of the bioactive compounds derived from plant sources is the extraction process. Modern extraction techniques, like ultrasound-assisted and supercritical fluid extraction methods, are currently used in addition to more conventional procedures [34]. Further, interviews with locals led for the identification of 27 plant species, mostly herbs but also shrubs and trees that were used to treat a variety of illnesses, with diarrhoea and gastric problems being the most common conditions. Curiously, the investigators discovered that more than half of these medical uses were unreported in the literature. Studies on ethnobotany often pay only sporadic attention to ornamental plants, focusing instead on plant species traditionally used for food or medicine. However, the widespread usage of a plant for ornamental purposes may also serve as the basis for research into additional possible uses. Pharmaceuticals, perfumes, flavours, and colourants account for a significant portion of the global industry for plant-derived chemicals, which is worth several billion dollars annually. Classic Taxol, vincristine, vinblastine, colchicine, the Chinese antimalarial drug artemisinin, and the Indian ayurvedic drug forskolin are a few examples of phytochemicals used in biology and medicine. The volume and exports of trade in medicinal plants are increasing. The annual value of the global trade in medicinal plants is estimated to be US$800 million.

For instance, around 1500 plant species from over 200 families and 800 genera have been processed into pharmaceuticals in Germany. Similarly, some 500 species are traded commodities in South Africa. Today, Poland, Germany, and Bulgaria are known as the top exporters of herbal medicines. The growth and commercialization of bioindustries based on medicinal plants in underdeveloped nations depend on the availability of resources and knowledge about upstream and downstream bioprocessing, extraction, purification, and marketing of the industrial potential of medicinal plants. Additionally, the lack of modernised socioeconomic and public healthcare systems increases the reliance of rural and low-income urban residents on the usage of traditional medicinal herbs and plants as supplementary aids to mainstream pharmaceutical market products. Without having undertaken in-depth research among numerous indigenous and other tribes, recent estimates show that over 9,000 plants have known medical benefits in various cultures and nations. The chemical structures of some potential natural compounds with MI activity are shown in Fig. (**1**).

## 2 METHODOLOGY

**Fig. (2):**
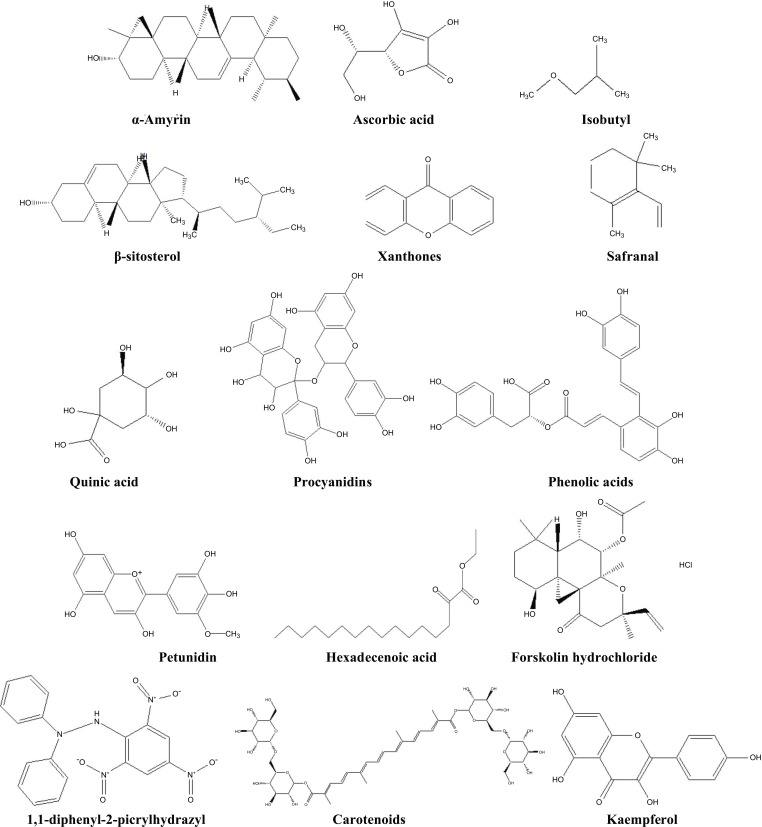
The chemical structure of some potential natural compounds with MI activity.

Google Scholar, Scopus, PubMed, and Web of Science were used to search the literature related to papers from the years 2001 to 2023, covering the knowledge regarding cardioprotective plants. Keywords like heart, cardioprotective and Indian medicinal system, *Hydrocotyle asiatica* and cardioprotective, cardioprotective plants and phytochemistry, cardioprotection and pharmacological properties *etc*., were included in the search. A total of 200 articles were retrieved, out of which 169 articles were considered. An effort was made to compile pertinent writings that were only concerned with cardioprotective plants. Until now, such scientific and molecular data have not been available in published articles. Thus, this review opens many new doors for the upcoming researchers and is helpful for the preparation of a monograph of the plant according to different medical systems (Fig. **2**).

## 3 RESULTS AND DISCUSSION

**Fig. (3):**
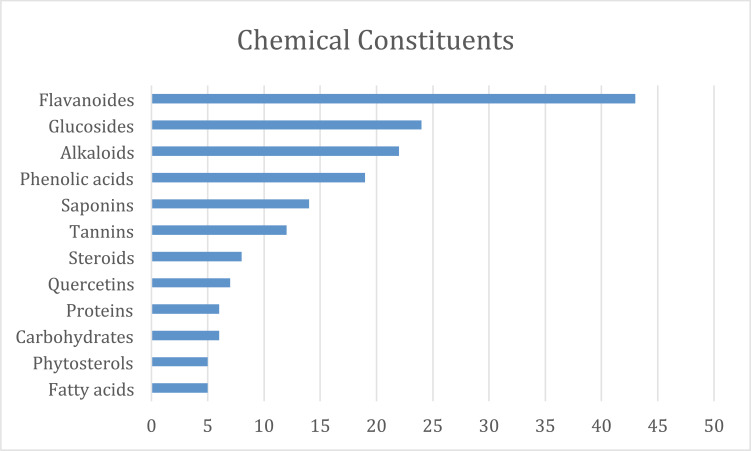
Chemical constituents.

**Fig. (4):**
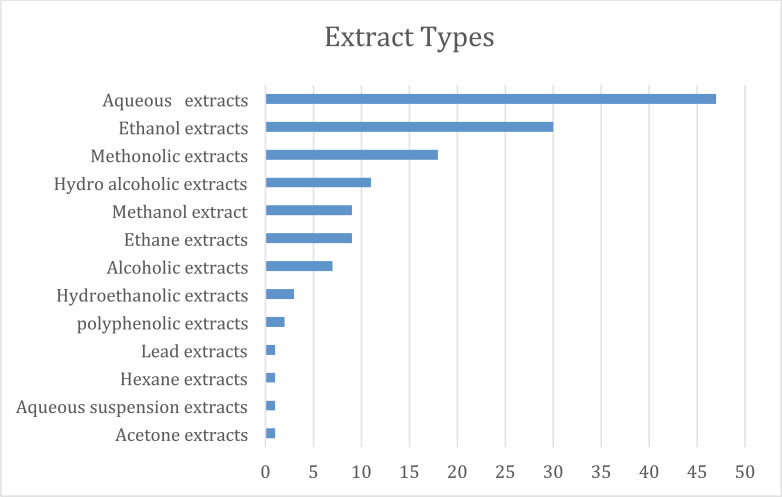
Plant extract types.

**Fig. (5):**
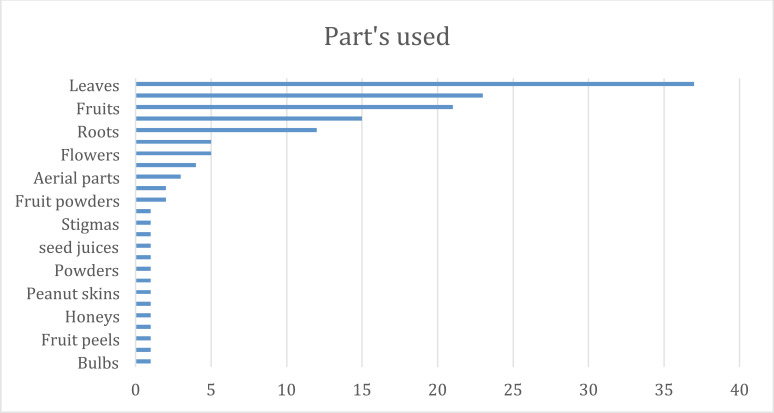
Plant parts used.

**Fig. (6):**
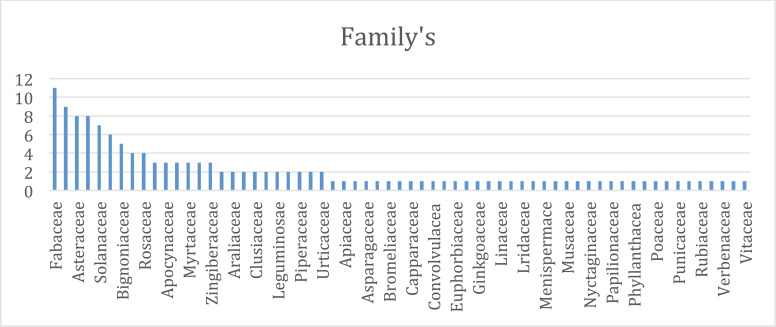
Plant family analysis.

Numerous botanicals have been found to have cardiac action (mainly from ethnopharmacological investigations; Table **1**). Nevertheless, most of the published literature has mostly concentrated on their pharmacological effects on experimental animals. Numerous studies have demonstrated that various herbs are utilized in conventional medicine to treat myocardial infarction. There have been several reports of natural products, such as flavonoids, alkaloids, saponins, tannins, *etc*, for the treatment of myocardial infarction. Among the most prevalent secondary metabolites found in the majority of plants, flavonoids are of special importance. All naturally occurring substances that were used in myocardial infarction were found to contain the following substances: flavonoids (43%), glucosides (25%), alkaloids (23%), phenolic acid (19%), saponins (15%), and tannins (12%). The remaining 12% of substances were classified as “other compounds” in this review and included carbohydrates, fatty acids, and sterols (Fig. **3**). According to an analysis of the plants employed, the extraction procedure of this review used leaves the most (37%), along with fruits, (23%), roots (20%), and flowers (5%), *etc*. (Fig. **4**). At the same time, the aqueous extracts (47%), ethanolic extracts (30%), and methanolic extracts were used (17%) (Fig. **5**). Nonetheless, it is advised to conduct proof-of-concept randomized controlled trials to confirm the efficacy and safety of these items in the clinical context given the encouraging findings of numerous medicinal plants and plant-derived phytochemicals against the myocardial infarction. Further, Fabaceae, Asteraceae, Solanaceae, and Bignoniaceae were the most prevalent plant families and were found in 8%, 4%, 3.5, and 1.5% of the research, respectively, according to an analysis of plant families (Fig. **6**).

## CONCLUSION

Several primary and secondary metabolites in plants are responsible for curing a wide range of acute and chronic ailments; however, many pharmacological actions exhibited by these plants have not been documented yet. Thus, with this study, the new researchers may conveniently carry out the tasks for the best plant exploration; they may also find a cure for ailments and open up new avenues for other researchers in the future.

## Figures and Tables

**Fig. (1):**
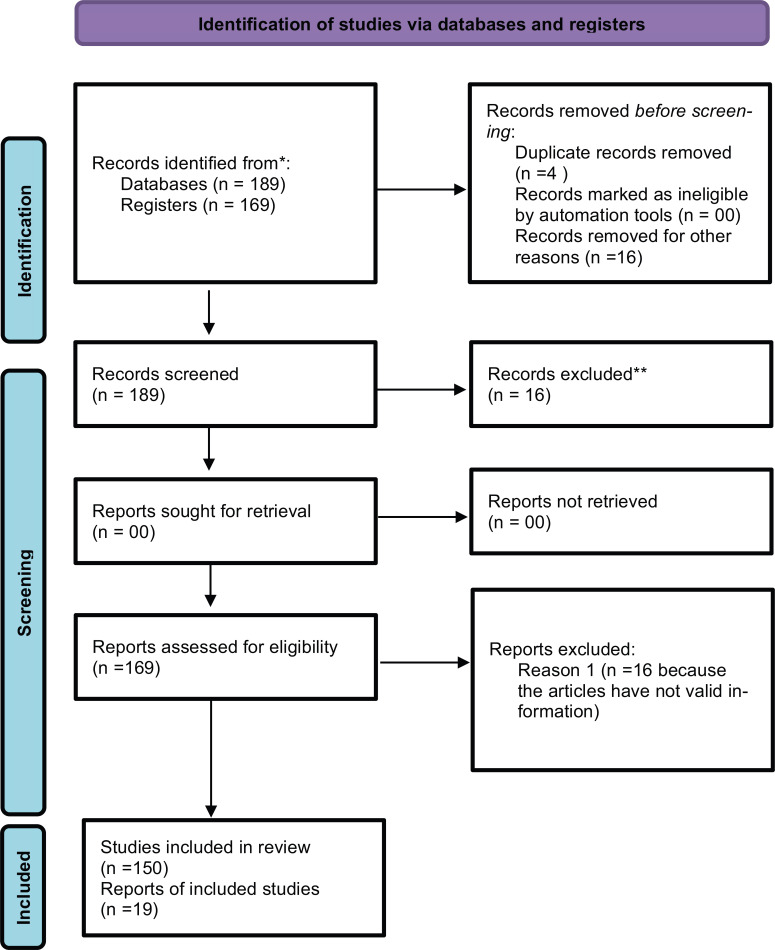
PRISMA 2020 flow diagram for new systematic reviews, which included searches of databases and registers only.

**Table 1:** Selection of the medicinal plants mentioned in this study.

**S. No**	**Plant** **Name**	**Common** **Name**	**Family**	**Part used**	**Chemical constituents**	**Extract type**	**Cadioprotective Model**	**Dose** **Mg/kg**	**Experimental Animals**	**References**
1	*Hydrocotyle asiatica L*	Centella asiatica	Araliaceae	Whole plant	Monoterpenoids sesquiterpenes	Alcoholic extract	Ischemia-reperfusion-induced myocardial infarction in rats	100-1000	Male wistar rats	[35]
2	*Solanum xanthocarpum*	Kantakari	Solanaceae	Leaves	Flavonoids, alkaloids	Hydro ethanolic	Isoproterenol (ISO) induced myocardial infarction	200	Adult female wistar rats	[36]
3	*Viscum album L*	European mistletoe	Loranthaceae	Leaves	Flavonoids, alkaloids	Aqueous extract	ISO-induced heart failure	250	Male wistar albino rat	[37]
4	*Boerhaavia diffusa Linn*	Punarnava	Nyctaginaceae	Whole plant	Flavonoids, alkaloids	Ethanolic extract	Doxorubicin-induced myocardial toxicity in albino rats	150-200	albino wistar rats	[38]
5	*Fuji apple*	Malus domestic	Rosaceae	Fruit pulp	Quercetin catechin	-	ISO-induced cardiotoxicity	100	Albino wistar rats	[39]
6	*Hibiscus rosa*	China rose	Malvaceae	Flower	Flavonoids, alkaloids	Aqueous Extract	Myocardial ischemic reperfusion injury in rats	250	Wistar albino rats	[40]
7	*Coriandrum sativum L.*	Cilantro	Apiaceae	Seeds	-	Methanolic Extracts	ISO-induced myocardial necrosis in rats	300	male wistar rats	[41]
8	*Psidium guajava L.*	Guava	Myrtaceae	Whole plant	Flavonoids carotenoids	Aqueous extracts	Reperfusion injury in perfused rats' hearts	250	Albino wistar rat	[42]
9	*Murraya koenigii (L.)*	Curry leaf	Rutaceae	Leaves	Flavonoids phenols	Aqueous extract	Doxorubicin-induced cardiotoxicity in rats	200	Female wistar albino rats	[43]
10	*Azadirachta indica*	Neem tree	Meliaceae	Leaves	Nimbolide limonoids	Aqueous Extract	Isoprenaline-induced myocardial infarction in rats	150-200	Male wistar rat	[44]
11	*Myrrh*	Commiphora myrrh	Burseraceae	Stem	Germacrene Curzerene	Water extract	ISO induced myocardial infarction in rats	50-100	Wister rats	[45]
12	*Chitosan- pinus merkusii*	Mindoro pine	Pinaceae	Whole plant	Phenolics alkaloid	Lead extract	Lead acetate-induced cardiac cell damage	100-200	Male wistar rat	[46]
13	*Sesamum indicum*	Sesame	Pedaliaceae	Seeds	Cerebrosides sitosterols	Aqueous extracts	Oxidative stress induced by cadmium in wistar rats	200	Male wistar albino rats	[47]
14	*Terminalia arjuna*	Arjun tree	Combretaceae	Bark	Annins, triterpenoid saponin, Flavonoids	Aqueous extracts	ISO Induced myocardial infarction	250	Adult wistar albino rats	[48]
15	*Lagenaria siceraria*	Bottle gourd	Cucurbitaceae	Fruit powder	Flavonoids, glycosides	Methanolic extract	ISO Induced myocardial infarction	500	Male wistar rats	[49]
16	*Curcuma longa*	Turmeric	Zingiberaceae	Rhizome	Curcuminoids	Ethanolic extract	Doxorubicin-induced cardiotoxicity in rats	200	Male sprague-dawely rats	[50]
17	*Citrullus colocynthis*	Colocynth	Cucurbitaceae	Fruits	Glycosides Flavonoids	Ethanol extract	Adrenaline-induced myocardial damage	100	Male rabbits	[51]
18	*Nerium oleander Linn*	Kanher	Apocynaceae	Flower	Gitoxigenin	Hydro- ethanolic extract	ISO-Induced Myocardial oxidative stress in experimental rats	100	Male sprague-dawley rat	[52]
19	*Allium sativum*	Garlic	Amaryllidaceae	Bulb	Allicin Diallyl	Aqueous extract	Oxidative stress induced by ischemic reperfusion injury	60	Male wistar rats	[53]
20	*Punica granatum*	Pomegranate	Punicaceae	Seed juice	Kaempferol	Aqueous extracts	ISO Induced myocardial infarction	100	Male adult albino wistar rats	[54]
21	*Hybanthus enneaspermus*	Blue spade flower.	Violaceae	Fruit	Flavonoids Alkaloids	Ethanolic extract	ISO Induced myocardial infarction	500	Male albino wister rats	[55]
22	*Gmelina arborea*	Gamhar	Verbenaceae	Leaves	Quercetin Apigenin	Ethanol extract	Doxorubicin-induced myocardial necrosis in albino rats	100	Wistar albino rats	[56]
23	*Glycyrrhiza uralensis*	Chinese liquorice	Fabaceae	Root	Flavonoids Stilbenoids	Hexane Extract	Doxorubicin-induced cardiotoxicity	100	Male mice	[57]
24	*Ficus racemosa Linn.*	Goolar	Moraceae	Bark	Flavonoids Alkaloids	Acetone extract o	Doxorubicin-induced cardiotoxicity	250-500	Male wistar rats	[58]
25	*Bombax ceiba L*	Silk cotton tree	Malvaceae	Flower	Quercetin Flavonoids	Aqueous extract	Acute adriamycin-induced myocardial infarction in rats	150-450	Male wistar rats	[59]
26	*Vitis* v*inifera*	Grapes	Vitaceae	Seeds	Flavonoids	alcoholic extract	ISO-induced myocardial necrosis	100	Wistar albino rats	[60]
27	*Tinospora cordifolia miers*	Gulbel	Menispermaceae	Whole plant	Glycosides Alkaloids	Alcoholic extract	Calcium chloride-induced cardiac arrhythmia in rats	150,250,450	Wistar albino rats	[61]
28	*Salvia miltiorrhiza*	Danshen	Lamiaceae	Root	Miltirone	Ethane Extract	ISO induced myocardial infarction	60	Sprague-dawley male rats	[62]
29	*Momordica dioica Roxb.*	Biiter gourd	Cucurbitaceae	Fruit	Flavonoids	Methanolic extract	Doxorubicin-induced cardiotoxicity	200	Albino rats	[63]
30	*Justicia traquebareinsis Linn*	Sivanervembu	Acanthaceae	Leaves	Flavonoids Glycosides	Aqueous extract	ISO induced myocardial infarction	100-200	Wistar strains of albino rats	[64]
31	*Bauhinia variegata*	Orchid tree	Fabaceae	Root	Quercetin Rutin	Aqueous extract	Cacl2-induced arrhythmia in albino rats	400	Wistar albino rats	[65]
32	*Cassia tora*	Senna tora	Fabaceae	Leaves	Glycosides Obtusifolin	Methanol extract	ISO-induced myocardial injury	100	Male wistar rats	[66]
33	*Arachis hypogaea*	Peanut	Fabaceae	Peanut skins	Flavonoids Alkaloids	Methanolic extract	ISO Induced myocardial infarction	30	Male & female albino rats	[67]
34	*Picrorrhiza kurroa*	Katuki	Plantaginaceae	Roots	Alkaloids	Hydroalcoholic extract	Induced cardiotoxicity in rats	200	Wistar albino rats	[68]
35	*Amaranthus viridis Linn*	Slender amaranth	Amaranthaceae	Whole plant	Flavonoids Alkaloids	Methanol extract	ISO induced myocardial infraction	100-200	Male wistar rats	[69]
36	*Stachys schimperi*	Hedgenettle	Lamiaceae	Aerial parts	C9-iridoidal glycosides,39 labdane, neo-clerodane,40 diterpenoids, flavonoids,41 phenylpropanoids	Methanolic extract	Doxorubicin -induced cardiotoxicity in rats	100	Male Wister rats	[70]
37	*Ananas comosus*	Pineapple	Bromeliaceae	Fruit	Ketones Aldehydes	Hydroalcoholic extract	ISO induced myocardial infraction	200-400	Albino wistar rats	[71]
38	*Pithecellobium dulce*	Manila tamarind	Leguminosae	Fruit peel	Ethyl gallate Catechin	Ethane extract	ISO induced myocardial infraction	200	Male wistar rats	[72]
39	*Carissa opaca*	Jungli karonda	Apocynaceae	Leaves	Ferric thiocyanate	Methanol extract	ccl4 induced cardiotoxicity in rats	100	Male sprague dawley rats	[73]
40	*Scleria lithosperma*	Slender nut rush	Cyperaceae	Whole plant	__	Ethanolic extract	Doxorubicin-induced cardiotoxicity in wistar albino rats	250-500	Wistar albino rats	[74]
41	*Sechium edule*	Chayote	Cucurbitaceae	Fruit	Trilinolenin Phenylacetic acid	Ethanolic extract	ISO-induced myocardial infarction	200	Albino wistar rats	[75]
42	*Nelumbo nucifera*	Sacred lotus	Nelumbonaceae	Leaves	Alkaloids Neferine	Alcoholic extract	ISO-induced cardiotoxicity in wistar rats	400	Adult male albino rats	[76]
43	*Garcinia indica*	Kokum	Clusiaceae	Fruits	Alkaloids	Hydroalcoholic extract	ISO-induced myocardial necrosis in rats	400& 800	Male wistar rats	[77]
44	*Fumaria indica*	Indian fumitory	Papaveraceae	Whole plant	Kernels contain 20 – 24% fatty oil	Methanol extract	ISO induced myocardial infraction	100	Albino wistar rats	[78]
45	*Hibiscus sabdariffa*	Roselle	Malvaceae	Petals	Quercetin Flavonoids	Aqueous extract	ISO induced myocardial infraction	300	Albino Wistar rats	[79]
46	*Kigelia africana*	Sausage tree	Bignoniaceae	Leaves	Flavonoid Polyphenols	Alcoholic Extract	ISO-induced myocardial injury	100-200	Male wistar rats	[80]
47	*Lavandula* *angustifolia*	English lavender	Lamiaceae	Flowers, aerial parts	Linalool Linalyl acetate	Ethanolic extract	ISO	100	Male wistar rats	[81]
48	*Solanum nigrum*	Black nightshade	Solanaceae	Stem & leaves	Flavonoids	Methanolic extract	Doxorubicin-induced cardiotoxicity	20	Adult wistar albino rats	[82]
49	*Moringa oleifera*	Moringa	Moringaceae	Leaves	Flavonoids alkaloid	Aqueous extract	ISO-induced cardiotoxicity in rats	40	Male albino rats	[83]
50	*Ocimum basilicum*	Basil	Lamiaceae	Seeds	Flavonoids Alkaloids	Methanolic extract	ISO-induced acute myocardial infarction in male rabbits	70	Male rabbits	[84]
51	*Potentilla reptans L.*	Creeping cinquefoil	Rosaceae	Root	Flavonoids	Aqueous extract	ISO-induced myocardial infarction	250	Male wistar rats	[85]
52	*Asparagus racemosus*	Shatavari	Asparagaceae	Root	Quercetin Sitosterol	Ethanol extract	Doxorubicin-induced cardiotoxicity in albino rats	250-500	Albino wistar rats	[86]
53	*Mangifera indica*	Mango	Anacardiaceae	Fruit	Flavonoids phenolic acids	Polyphenolic extracts	Doxorubicin-induced cardiotoxicity in albino rats	300	Sprague dawley strain albino rats	[87]
54	*Aconitum* *carmichaelii*	Aconite	Ranunculaceae	Roots	Neoline, ephedrine, sparteine, picraconitine, acotinic acid	Aqueous extract	ISO-induced myocardial infarction	100	Albino male wistar rats	[88]
55	*Pterocarpus* *mildbraedii*	Mukwa	Fabaceae	Leaves	-	Methanolic extract	ISO-induced myocardial infarction in rats	100	Male albino wistar rats	[89]
56	*Datura metel*	Horn of plenty	Solanaceae	Seeds	Alkaloids Glycosides	Aqueous extract	Acute cardiotoxicity induced with doxorubicin in wistar rats	100,200,300	Adult male wistar rats	[90]
57	*Trigonella* *foenum – graecum*	Fenugreek	Fabaceae	Whole plant	Alkaloids Steroids	Ethanol extract	ISO-induced myocardial infarction	400	adult wistar albino rats	[91]
58	*Musa paradisiaca*	Banana	Musaceae	Peels	Tannins Flavonoids	Aqueous extract	ISO-induced myocardial infarction rat model	10 &100	Male wistar rats	[92]
59	*Urtica simensis*	Burn nettle	Urticaceae	Leaves	Flavonoids tannins	Aqueous extract	Cyclophosphamide-induced myocardial injury in rats	400	Albino wistar rat	[93]
60	*Malva verticillata*	Chinese mallow	Malvaceae	Leaves	Alkaloids Phenols	Methanolic extract	Doxorubicin-induced cardiotoxicity in rats	50	Male albino rats	[94]
61	*Citrullus colocynthis Linn*	Colocynth	Cucurbitaceae	Fruits	Flavonoids alkaloids	Ethane extract	ISO induced myocardial infraction	100	Male rabbits	[51]
62	*Indigofera barberi*	Sinnichedi	Fabaceae	Whole plant	Tannins glycosides	Ethanol extract	Doxorubicin-induced toxicity on rats	250-500	Male wister rats	[95]
63	*Randia dumetorum*	Madanaphala	Rubiaceae	Fruits	Flavonoids saponins	Ethanolic extract	Doxorubicin-induced cardiotoxicity in rats	200-400	Male swiss albino mice	[96]
64	*Cassia fistula L.*	Golden shower	Fabaceae	Bark	Glycerides Resinous	Aqueous extracts	ISO-induced myocardial infarction in rat	250	Male and female albino wistar rats	[97]
65	*Rumex vesicarius Linn*	Bladder dock	Polygonaceae	Leaves	Tannins Flavonoids	Aqueous extract	ISOinduced myocardial infarction	1000,200,300	Male rabbits	[98]
66	*Vitex negundo*	Chastetree	Lamiaceae	Leaves	Sesquiterpenes, lignan, flavonoids, flavones, glycosides	Ethanolic extract	ISO-induced myocardial necrosis in wistar rats	300	Albino rats of Wistar strain	[99]
67	*Alpinia zerumbet*	Shell flower	Zingiberaceae	Leaves	Α-terpineol	Hydro alcoholic extract	ISO-induced myocardial infarction in rats	300	Male wistar rats	[100]
68	*Sour cherry*	Prunus cerasus	Rosaceae	Seed	Β-carotene Tocopherols	Aqueous extract	ISO-induced myocardial infarction	250	Rabbit heart	[101]
69	*Syzygium* *aromaticum*	Clove	Myrtaceae	Whole plants	Flavonoids Βesquetrepene ester	Methanolic extract	ISO-induced myocardial infarction in rats	250,500,750	Male wistar rats	[102]
70	*Newbouldia laevis*	Boundary tree	Bignoniaceae	Leaf and root	Tannins Flavonoids	Aqueous extract	ISO-induced myocardial infarction	800	Male albino rats	[103]
71	*Linum* *usitatissimum*	Flax	Linaceae	Seeds	Alkaloids Lignans	Methanol extract	Acute myocardial infarction in male rabbits induced by isoproterenol	50	male domestic rabbits	[84]
72	*Cinnamomum tamala*	Tejpata	Lauraceae	Leaves	Glycosides, saponins, carbohydrates, sterols, alkaloids, Flavonoids, tannins, proteins and triterpenoids	Aqueous extract	Doxorubicin-induced myocardial infarction	200-400	Wistar albino rats	[104]
73	*Lepidium sativum*	Garden cress	Brassicaceae	Seeds	Flavonoids sapogenin	Water extract	Against 5-fu-induced cardiotoxicity and oxidative stress in albino rats	550	Adult male albino sprague–dawley rats	[105]
74	*Tamarindus indica Linn*	Tamarind tree	Caesalpiniaceae	Fruit	Phenols Glycoside	Aqueous & alcoholic extract	ISO-induced myocardial infarction	250-500	Albino wistar rats	[106]
75	*Raphanus sativus Linn*	Radish	Brassicaceae	Leaves	Alkaloids, glycosides, saponins, phytosterol, flavonoids, phenols,	Aqueous extract	ISO-induced injury to myocardial cells includes hypoxia	500	Adult male albino rat	[107]
76	*Carthamus* *tinctorius*	Safflower	Asteraceae	Seed	Phenolics, flavonoids, alkaloids, lignans, carboxylic acids	Ethanolic extract	ISO-induced myocardial infarction	200	Adult rats.	[108]
77	*Ganoderma lucidum*	Reishi	Ganodermataceae	Whole plant	Triterpenoids, alkaloids, steroids, polysaccharides	Aqueous extracts	ISO-induced myocardial infarction	400	male wistar rats	[109]
78	*Alstonia scholaris*	Echites scholaris	Apocynaceae	Dried bark	Alkaloids, carbohydrates, glycosides, saponins, phytosterols, tannins, flavonoids,	Ethanolic extract	ISO-induced myocardial infarction rats	200-400	Albino wistar rats	[110]
80	*Abelmoschus* *esculentus*	Okra	Malvaceae	Fruit	Flavonioid glycosides isoquercetin and quercetin-3-o	Aqueous crude extract	ISO-induced myocardial infarction	50-100	Albino Wistar rat	[111]
81	*Aloe vera*	Aloe vera	Asphodelaceae	Leaves	Phenol–sulfuric acid M-hydroxydiphenyl method	__	oxidative stress-induced myocardial apoptosis	100	Albino Wistar rats	[112]
82	*Brassica oleracea*	Broccolis	Brassicaceae	stem	Glucosinolates, dithiolthiones, indoles, s-methyl cysteine sulfoxide, isothiocyanates, indole-3-carbinol	Aqueous extract	Ischaemia reperfusion-induced cardiac injury	80	Sprague–Dawley male rats	[113]
83	*Spinacia oleracea*	Spinach	Amaranthaceae	Leaves	Flavonoids, quercetin and rutin	Aqueous extract	ISO-induced myocardial infarction in rats.	500	Male Wistar albino rats.	[114]
84	*Ficus hispida Linn*	Kannada	Moraceae	Leaf	Flavonoid Tannins	Methanol extract	Cyclophosphamide provoked oxidative myocardial injury in rat	400	Male wistar rat	[115]
85	*Urtica parviflora*	Sishnu	Urticaceae	Leaves	Alkaloids, glycosides, Phenolic compounds and flavonoids	Ethanolic extract	ISO-induced myocardial infarction in rats	350	Albino rats of Sprague-Dawley strain	[116]
86	*Inula racemosa*	Pushkarmool	Asteraceae	Roots	Inulin, alantolactone, β-sitosterol, isoalantolactone, dihydroalantolactone, and glycosides	Hydroalcoholic extract	ISO-induced myocardial infarction in rats	100	Adult wistar rats	[117]
87	*Leptospermum scoparium*	Honey	Apidae	Honey	1.13% proteins, 0.36% minerals, 215.2 mg/g lipid, 15.5 mg/kg (hydroxymethyl)furfural	-----	ISO-induced myocardial injury	200	Male wistar albino rats	[118]
88	*Malva sylvestris*	Mallow	Malvaceae	Whole plant	Anthocyanidines, coumarins	methanolic extract	myocardial injury in I/R-induced myocardial infarction	250	male albino rats	[119]
89	*Astragali radix*	Astragalus	Fabaceae	Fruits	Flavonoids, saponins, polysaccharides, amino acids, and trace elements	Aqueous extract	ISO-induced myocardial injury	20,10	Male Sprague-Dawley rats	[120]
90	*Acacia senegal*	Gum arabic	Fabaceae	Seeds	Glacial acetic acid, hydrogen peroxide,	Ethane extract	ISO-induced myocardial infarction	500	Rabbit	[121]
91	*Wedelia* *calendulacea*	Creeping-oxeyes	Asteraceae	Stem	Glutathione 5-5′-dithiobis-2-nitrobenzoic acid	Aqueous extract	ISO-induced myocardial injury	250	Male wistar rats	[122]
92	*Chichorium intybus*	Chicory	Asteraceae	Whole plant	Flavonoid	Aqueous extract	ISO-induced myocardial infarction	100	Male wistar rats	[123]
93	*Amaranthus tricolor*	Red spinach	Amaranthaceae	Leaves	Β-carotene, phenolic acids, flavonoids	Methanolic extract	Isoprenaline-induced myocardial damage	50	wistar female rats	[124]
94	*Achillea wilhelmsii*	Yarrow	Asteraceae	Whole plant	Α-thujene (6.11%), α-pinene (5.11%), sabinene (5.23%), p-cymene (7%), 1,8-cineole (6%), linalool (10%),	Ethane extract	Reperfusion-induced injury to the myocardium	200	Male wistar rats	[125]
95	*Populus ciliata*	Himalayan poplar	Salicaceae	Stem and leaves	Hexadecenoic acid, ethyl ester As corbic acid,	Ethanolic & aqueous	ISO-induced acute myocardial infarction	250	Albino rabbits& sprague dawley albino rats	[126]
96	*Trichosanthes*	Snake gourd	Cucurbitaceae	Whole plant	Proteins, fat, fibre, carbohydrates, vitamin a and e	Methanol extract	Doxorubicin-induced cardiotoxicity in rats	500	male wistar rats	[127]
97	*Ocimum sanctum*	Holy basil	Lamiaceae	Leaves	Phenolic compounds apigenin and rosameric	Hydroalcoholic extract	ISO-induced myocardial infarction	200	Wistar Albino rats	[128]
98	*Syzygium cumini*	Malabar plum	Myrtaceae	Seed	Astragalin, kamepferol-3-0- glucoside, myricetin, and gallic acid.	Methanolic extract	Glucose-induced oxidative stress in h9c2 cardia	100	Albino wistar rat	[129]
99	*Trichosanthescucumerina*	Snake gourd	Cucurbitaceae	Fruit	Cucurbitacin b, cucurbitacin e, isocucurbitacin b, 23, 24 -dihydroisocucurbitacin b, 23,24-dihydrocucurbitacin e, sterols 2 β-sitosterol, stigmasterol, α-carotene	Methanol extract	Doxorubicin-induced cardiotoxicity in wistar rats	500	Male wistar rats	[127]
100	*Sesbania grandiflora*	Katurai	Fabaceae	Leaves	Adenine dinucleotide phosphate, ascorbic acid, and	Aqueous suspension	Cigarette smoke-induced oxidative damage	1000	Male wistar rats	[130]
101	*Semecarpus* *anacardium*	Varnish tree	Anacardiaceae	Nuts	Alkaloids, carbohydrates, glycosides, phytosterols, proteins, saponins, tannins and flavonoids.	Hydroalcoholic extract	ISO-induced myocardial damage in rat	100	Sprague-dawley rats	[131]
102	*Scutellaria radix*	Huang qin	Lamiaceae	Whole plant	Flavonoids	Aqueous extract	ISO-induced myocardial infarction rat	50	Male wistar rats	[132]
103	*Tridax procumbens linn*	Dhaman grass	Asteraceae	Whole plant	Flavonoids glycoside and quercetin	Hydro-alcoholic extract	ISO-induced myocardial infarction	100-200	male Wistar albino rats	[133]
104	*Oroxylum indicum*	Indian trumpet tree	Bignoniaceae	Whole plant	Flavonoids, glycoside, and volatile oil.	Aqueous alcohol extract	Doxorubicin-induced cardiotoxicity	250	Male mice	[134]
105	*Solanum* *lycopersicum*	Tomato	Solanaceae	Fruit	Flavonoids	Aqueous extract	ISO-induced myocardial infarction	10	Male adult albino rats	[135]
106	*Piper guineense*	Uziza	Piperaceae	Seeds	Isobutyl, pyrrolidyl and piperidyl amidealkaloids	Methanol extract	ISO-induced myocardial infarction	125-250	Wistar rats	[136]
107	*Cucumis trigonus*	Thummittikai	Cucurbitaceae	Fruits	Phenolics Flavonoids, glycosides, and alkaloids	Ethanol extract	ISO-induced myocardial infarction	200	Male albino sprague dawely rats	[137]
108	*Evolvulus alsinoides*	Vishnukranti	Convolvulaceae	Whole plant	Scopoletin, umbelliferone, scopolin and 2-methyl-1,2,3,4-butanetetrol.	Methanolic extract	ISO-induced myocardial ischemic injury	100-200	Male Wistar rats	[138]
109	*Elettaria* *cardamomum*	Cardamom	Zingiberaceae	Fruit	Steroids, flavonoids, phenolics, amino acids, and alkaloids.	aqueous extract	ISO-induced myocardial infarction in rats	100	Wistar male albino rats	[139]
110	*Gynura procumbens*	Moluccan spinach	Asteraceae	Whole plant	Steroids caffeoylquinic acids, fatty acids, flavonoids, and terpenoid	Ethane extract	ISO-induced myocardial infarction	250	Male sprague-dawley rats	[140]
111	*Cynodon dactylon*	Cynodon dactylon	Poaceae	Rhizomes	Flavonoids, sterols, steroidal saponins and alkaloids	Hydroalcoholic extract	Ischemia/reperfusion-induced arrhythmias	50	Male sprague-dawley rats	[141]
112	*Momordica cymbalaria fenzl*	Hook	Cucurbitaceae	Roots	Flavonoids	Ethanolic extract	ISO-induced myocardial injury	250	male wistar rats	[142]
113	*Tecoma Stans*	Yellow trumpetbush	Bignoniaceae	Flower	Triterpenoids Phenolics P-sitosterol	ethanolic extract	ISO-induced myocardial infarction in rat myocardium	250-500	Wister albino rats	[143]
114	*Garcinia* *pedunculata*	Bor thekera	Clusiaceae	Fruit	Alkaloids, flavonoids	Aqueous extract	Isoprenaline-induced myocardial infarction in rat	200	Male wistar albino rats	[144]
115	*Dialium guineense*	Black tamarind	Leguminosae	Stem bark	4.0 g moisture, 8.3 g crude protein, 4.9 g crude lipids, 3.2 gash, 0.6 g crude fibre	Ethane and aqueous extract	Cardiac injury was induced with ccl4	1000	Adult male wistar rats,	[145]
116	*Phyllanthus urinaria L*	Chamberbitter	Phyllanthaceae	Whole part	Brevifolincarboxylic acid(vi), isostrictiniin(ix), geraniin (x), gallic acid(xi), and ellagic acid (xii).	Ethanolic extract	oxidative stress induced by ischemic reperfusion injury.	100	Wistar rat	[146]
117	*Piper betle*	Betel vine	Piperaceae	Leaves	Alkaloids, flavonoids, carbohydrates, tannins and terpenes	Aqueous extract	ISO-induced myocardial infarction	50	Male wistar albino rats	[147]
118	*Croton sparciflorus*	Astraea lobata	Euphorbiaceae	Whole plant	Alkaloids, flavonoids, phenols, tannins, saponin and terpenoids	Methanolic extract	ISO-induced myocardial infarction wistar albino rats	100	Male wistar albino rats n	[148]
119	*Cyperus rotundus*	Purple flat sedge	Cyperaceae	Tubers	Flavonoids, terpenoids, sesquiterpenes, sitosterol, cyperene, cyperol, nootkatone, and valencene	polyphenolic extract	ISO-induced myocardial infarction	15& 30	adult female rats (Rattus norvegicus)	[149]
120	*Buchanania axillaris*	Cuddapah almond	Anacardiaceae	Whole plant	Beta-sitosterol, quercetin,	Ethanolic extract	Doxorubicin-induced cardiotoxicity	500	Adult Wistar albino rats w	[150]
121	*Capparis divaricata Lam*	Spreading caper	Capparaceae	Leaves	_______	Ethanol extract	Doxorubicin -induced cardiac injury in rats	200	Male wistar albino rats	[151]
122	*Limonia acidissima*	Wood apple		Fruit	Alkaloids, flavonoids, steroids, saponins, glycosides, phenols, gum and mucilage, fixed oils and fats, resins and tannins.	Ethanolic extract	ISO-induced myocardial infarction	200	Healthy male Sprague–Dawley rats	[152]
123	*Hyoscyamus niger Linn*	Henbane	Solanaceae	Seed	-	Alcoholic extract	ISO-induced myocardial necrosis	100	Male albino rats	[153]
124	*Erythrina stricta*	Indian Coral tree	Papilionaceae	Leaves	Alkaloids	Ethanol extract	ISO-induced myocardial infarction	200	Wistar albino rats	[154]
125	*Lycium chinense miller*	Wolfberry	Solanaceae	Fruit	1,1-diphenyl-2-picrylhydrazyl (dpph) radical, nacetylcysteine (nac),	Methanol extract	Against oxidative stress-induced hepatotoxicity	500	young male rats	[155]
126	*Ginkgo biloba*	Ginkgo	Ginkgoaceae	Leaves	Flavonoid glycosides (kaempferol, quercitin, and isorhamnetin),	Ethane extract	ISO-induced myocardial necrosis in rats	100	Wistar albino rats	[156]
127	*Beta vulgaris*	Beetroot	Amaranthaceae	Leaves	Carotenoids, phenolic acids and flavonoids	Aqueous extract	Doxorubicin-induced cardiac injury	200	male albino wistar rats	[157]
128	*Aegle marmelos*	Bael	Rutaceae	Leaves	Alkaloids, carbohydrates, glycosides, phytosterols, proteins, saponins, tannins and flavonoids	Methanolic extract	ISO-induced myocardial damage in rats.	100	Female sprague-dawley rats	[158]
129	*Aralia elata*	Japanese angelica tree	Araliaceae	Root barks	Triterpene saponins	Ethane extract	Myocardial ischemic reperfusion injury in rats	100	Male wistar rats	[159]
130	*Silybum marianum*	Milk thistle	Asteraceae	Fruit, seeds	Flavonoid Lignans, phenolics	Aqueous extract	Ischemia-reperfusion-induced myocardial infarction in albino rats	250	Albino wistar rats	[160]
131	*Caesalpinia bonducella*	Bonduc nut	Caesalpiniaceae	Aerial parts	Isoflavones, steroidal saponin, fatty acids, hydrocarbons, amino acids, phenolics, and phytosterols	Aqueous plant	Doxorubicin-induced myocardial infarction in albino rats	150	Wistar strains of albino rats	[161]
132	*Citrus reticulate.*	Kinnow	Rutaceae	Fruit	Limonoids, flavonoids, phenolics, and flavonoids	Ethanol extract	Doxorubicin-induced myocardial infarction	300	Sprague Dawley strain albino rats	[87]
133	*Withania somnifera*	Ashwagandha	Solanaceae	Roots	Alkaloids Saponins	Hydro-alcoholic extract	ISO-induced myocardial necrosis in rats	50	Wistar albino male rats	[162]
134	*Spathodea* *campanulata*	African tulip tree	Bignoniaceae	Bark	Steroids, cardiac glycosides, flavonoids, tannins and polyphenols	Ethanolic extract	ISO-induced myocardial infarction	250	Wistar albino rats	[163]
135	*Trichopus zeylanicus*	Kerala ginseng	Dioscoreaceae	Leaves	_	Ethanol extract	ISO-induced myocardial infarction in rats.	500	Wister strain male albino rats	[164]
136	*Andrographis paniculata*	Kalmegh	Acanthaceae	Leaves	Andrographolide, diterpenoids, flavonoids, quinic acid, xanthones, noriridoids, and andrographidoid a, b, c, d, and e	Ethanol extract	ISO-induced myocardial infarction in rats	200	Male wistar rats	[165]
137	*Crocus sativus L.*	Saffron	Iridaceae	Stigma	Carotenoid compounds, crocetin, crocin, safranal, glucoside picrocrocin, anthocyanins, delphinidin, petunidin	Aqueous extract	ISO-induced myocardial infarction in wistar rats	80-160	Male wistar albino rats	[166]
138	*Coleus forskohlii*	Plectranthus barbatus	Lamiaceae	Roots	Forskolin hydrochloride, demethylcryptojaponol, α-amyrin, betulic acid, glycosides, forskolin	Ethanolic extract	Isoprenaline-induced myocardial infarction in rats	50	Wistar albino rats	[167]
139	*Crataegus oxycantha*	Hawthorn	Rosaceae	Seeds	Flavonoids, oligomeric procyanidins, triterpenes, phenolic acids, fatty acids, and sterols	Methanolic extract	ISO-induced myocardial injury.	100	ischemia/reperfusion (I/R)-induced arrhythmias	[168]
140	*Bacopa monniera*	Brahmi	Plantaginaceae	Whole plant	Bacosides, Hersaponin	Hydro-alcoholic extract	ISO-induced myocardial necrosis	150	Wistar albino rat	[169]

## Data Availability

The data and supportive information are available within the article.

## LIST OF ABBREVIATIONS

MI: Myocardial Infarction
T2MI: Type 2 Myocardial Infarction
T1MI: Type 1 Myocardial Infarction
CAD: Coronary Artery Disease
STEMI: ST-Elevation Myocardial Infarction

## STANDARDS OF REPORTING

PRISMA guidelines and methodology were followed.

## SUPPLEMENTARY MATERIAL

PRISMA checklist is available as supplementary material on the publisher’s website along with the published article. Supplementary material is available on the publisher’s website along with the published article.
